# Supplementary material for: In-situ glial cell-surface proteomics identifies pro-longevity factors in Drosophila
Source: eLife. 2026 Aug 4;14:RP109422. doi: 10.7554/eLife.109422 (PMC13436962; doi:10.7554/eLife.109422)
Supplement: Peer review history

# Editor report: eLife Assessment

**DOI:** 10.7554/eLife.109422.3.sa0

**Authors:** Yan Song

**Affiliations:** Peking University China

**Keywords:** Compelling, Fundamental

Combining state-of-art in-situ cell-surface proteomics, functional genetic screening, and single-nucleus RNA sequencing, this **fundamental** work substantially advances our understanding of glial contributions to organismal lifespan. The evidence supporting the conclusions is **compelling**. The work will be of broad interest to researchers studying aging biology, glia-neuron communication and in vivo proteomic profiling.

# Referee report: Reviewer #1 (Public review)

**DOI:** 10.7554/eLife.109422.3.sa1

Summary:

Age-related synaptic dysfunction can have detrimental effects on cognitive and locomotor function. Additionally, aging makes the nervous system vulnerable to late-onset neurodegenerative diseases. This manuscript by Marques et al. seeks to profile the cell surface proteomes of glia to uncover signaling pathways that implicated in age-related neurodegeneration. They compared the glial cell-surface proteomes in the central brain of young (day 5) and old (day 50) flies and identified the most up- and down-regulated proteins during the aging process. 48 genes were selected for analysis in a lifespan screen, and interestingly, most sex-specific phenotypes. Among these, adult-specific pan-glial DIP-β overexpression (OE) significantly increased the lifespan of both males and females and improved their motor control ability. To investigate the effect of DIP-β in the aging brain, Marques et al. performed snRNA-seq on 50-day old Drosophila brains with or without DIP-β OE in glia. Cortex and ensheathing glia showed the most differentially expressed genes. Computational analysis revealed that glial DIP-β OE increased the cell-cell communication, particularly with neurons and fat cells.

Strengths:

(1) State-of-the-art methodology to reveal the cell surface proteomes of glia in young and old flies.

(2) Rigorous analyses to identify differentially expressed proteins. 3

(3) Examination of up- and down-regulated candidates and identification of glial-expressed mediators that impact fly lifespan.

(4) Intriguing sex-specific glial genes that regulate life span.

(5) Follow-up RNA-seq analysis to examine cellular transcriptomes upon overexpression of an identified candidate (DIP-β).

(6) A compelling dataset for the community that should generate extensive interest and spawn many project.

Weaknesses:

(1) DIP-β OE using flySAM:

a) These flies showed a larger increase in lifespan compared to using UAS-DIP-β (Figure 2 C,D). Do the authors think that flySAM is a more efficient way of OE than UAS? Also, the UAS construct would be specific to one DIP-β isoform while flySAM likely would likely express all isoforms. Could this also contribute to the phenotypes observed?

b) The Glial-GS>DIP-β flySAM flies without RU-486 have significantly shorter lifespans (Figure 2C) than their UAS-DIP-β counterparts. flySAM is lethal when expressed under the control of tubulin-GAL4 (Jia et al. 2018) likely due to toxicity of such high levels of overexpression. Is it possible that larger increase in lifespan is due to the already reduced viability of these flies?

c) Statistics: It is stated in the Methods that "statistical methods used are described in the figure legend of each relevant panel." However, there is no description of the statistics or sample sizes used in Figure 2.

(2) Figure 3: The authors use a glial GeneSwitch (GS) to knock down and overexpress candidate genes. In Figure 3A, they look at glial-GS>UAS-GFP with and without RU. Without RU, there is no GFP expression, as expected. With RU, there is GFP expression. It is expected that all cell body GFP signal should colocalize with a glial nuclear marker (Repo). However, there is some signal that does not appear to be glia. Also, some many glia do not express GFP, suggesting the glial GS driver does not label all glia. This could impact which glia are being targeted in several experiments.

(3) It is interesting that sex-specific lifespan effects were observed in the candidate screen.

a) The authors should provide a discussion about these sex-specific differences and their thoughts about why these were observed.

b) The authors should also provide information regarding the sex of the flies used in the glial cell surface proteome study.

c) Also, beyond the scope of this study, examining sex-specific glial proteomes could reveal additional insights into age-related pathways affecting males and females differentially.

(4) The behavioral assay used in this study (climbing) tests locomotion driven by motor neurons. The proteomic analysis was performed with the central adult brain, which does not include the nerve cord where motor neurons reside. While likely beyond the scope of this study, it would be informative to test other behaviors including learning, circadian rhythms, etc.

(5) It is surprising that overexpressing a CAM in glia has such a broad impact on the transcriptomes of so many different cell types. Could this be due to DIP-β OE maintaining the brain in a "younger" state and indirectly influencing the transcriptomes? Instead of DIP-β OE in glia directly influencing cell-cell interactions? Can the authors comment on this?

Comments on revisions:

The authors have conducted additional experiments, updated text/figures, and included discussions to address the concerns raised by the reviewers. I commend the authors on a thorough, rigorous study that will undoubtedly impact the field and spawn many projects for years to come.

One minor comment: In Figure S2, the figure legend states "A-C"; however, the figure itself only has an A and B.

# Author comment: Author response

**DOI:** 10.7554/eLife.109422.3.sa2

**Authors:** Madeline P Marques, Bo Sun, Ye-Jin Park, Tyler Jackson, Tzu-Chiao Lu, Yanyan Qi, Erin Harrison, Miranda C Wang, Omar Moussa Pasha, Amogh Varanasi, Dominique Kiki Carey, DR Mani, Jonathan Zirin, Mujeeb Qadiri, Yanhui Hu, Kartik Venkatachalam, Norbert Perrimon, Steven A Carr, Namrata D Udeshi, Liqun Luo, Jiefu Li, Hongjie Li

**Affiliations:** Baylor College of Medicine Houston United States; Baylor College of Medicine Houston United States; Baylor College of Medicine Houston United States; Baylor College of Medicine Houston United States; Baylor College of Medicine Houston United States; Baylor College of Medicine Houston United States; Baylor College of Medicine Houston United States; Baylor College of Medicine Houston United States; Baylor College of Medicine Houston United States; Baylor College of Medicine Houston United States; Broad Institute Cambridge United States; Broad Institute Cambridge United States; Harvard Medical School Boston United States; Harvard Medical School Boston United States; Harvard Medical School Boston United States; McGovern Medical School at the University of Texas Health Sciences Center (UTHealth) Houston United States; Harvard University Boston United States; Broad Institute Cambridge United States; Broad Institute Cambridge United States; Howard Hughes Medical Institute, Stanford University Stanford United States; Janelia Research Campus Ashburn United States; Baylor College of Medicine Houston United States

The following is the authors’ response to the original reviews

**Public Reviews:**

**Reviewer #1 (Public review):**

Summary:

Age-related synaptic dysfunction can have detrimental effects on cognitive and locomotor function. Additionally, aging makes the nervous system vulnerable to late-onset neurodegenerative diseases. This manuscript by Marques et al. seeks to profile the cell surface proteomes of glia to uncover signaling pathways that are implicated in age-related neurodegeneration. They compared the glial cell-surface proteomes in the central brain of young (day 5) and old (day 50) flies and identified the most up- and down-regulated proteins during the aging process. 48 genes were selected for analysis in a lifespan screen, and interestingly, most sex-specific phenotypes. Among these, adult-specific pan-glial DIP-β overexpression (OE) significantly increased the lifespan of both males and females and improved their motor control ability. To investigate the effect of DIP-β in the aging brain, Marques et al. performed snRNA-seq on 50-day-old Drosophila brains with or without DIP-β OE in glia. Cortex and ensheathing glia showed the most differentially expressed genes. Computational analysis revealed that glial DIP-β OE increased cell-cell communication, particularly with neurons and fat cells.

Strengths:

(1) State-of-the-art methodology to reveal the cell surface proteomes of glia in young and old flies.

(2) Rigorous analyses to identify differentially expressed proteins.

(3) Examination of up- and down-regulated candidates and identification of glial-expressed mediators that impact fly lifespan.

(4) Intriguing sex-specific glial genes that regulate life span.

(5) Follow-up RNA-seq analysis to examine cellular transcriptomes upon overexpression of an identified candidate (DIP-β).

(6) A compelling dataset for the community that should generate extensive interest and spawn many projects.

Weaknesses:

(1) DIP-β OE using flySAM:

(a) These flies showed a larger increase in lifespan compared to using UAS-DIP-β (Figure 2 C, D). Do the authors think that flySAM is a more efficient way of OE than UAS? Also, the UAS construct would be specific to one DIP-β isoform, while flySAM would likely express all isoforms. Could this also contribute to the phenotypes observed?

We agree with the reviewer that both can contribute to the different lifespan effect. In the original paper presenting flySAM1.0 and flySAM 2.0 (Jia et al., 2018), the authors first tested how flySAM1.0 overexpression (OE) phenotypes compare to several VPR (CRISPRa) and UAS:cDNA OE lines. They found that flySAM1.0 reliably outperforms (i.e., produces stronger OE phenotypes) than VPR in most cases, and produces OE phenotypes that are comparable (i.e., generally equivalent) to UAS:cDNA (Jia et al., 2018). After determining how flySAM1.0 performance compares to VPR and UAS:cDNA, the authors next tested if flySAM2.0 also outperforms VPR; they found that like flySAM1.0, flySAM2.0 outperforms VPR in most cases (Jia et al., 2018). In general, the data suggest that we should expect comparable overexpression phenotypes for our flySAM2.0 and UAS:cDNA lines.

We chose to proceed with the *DIP-β* flySAM line for the climbing assays and snRNA-seq, as it gave a stronger lifespan effect and we thought it was likely to be the more robust OE line. While our glial cell-surface proteomics initially identified DIP-β isoform C as the candidate, it is possible that other DIP-β isoforms were also present (such as isoform F, which is identical in polypeptide sequence to isoform C) (FlyBase). Ultimately, we believe that the larger increases in lifespan observed for DIP-β flySAM are likely because flySAM targets all isoforms, whereas UAS:cDNA lines target only one isoform. Importantly, our UAS- DIP-β line was specific to DIP-β isoform C, which is the same isoform that was identified by our proteomics.

We have made clarifications in the manuscript to address these comments.

(b) The Glial-GS>DIP-β flySAM flies without RU-486 have significantly shorter lifespans (Figure 2C) than their UAS-DIP-β counterparts. flySAM is lethal when expressed under the control of tubulin-GAL4 (Jia et al. 2018), likely due to the toxicity of such high levels of overexpression. Is it possible that a larger increase in lifespan is due to the already reduced viability of these flies?

This is a good point. The flySAM lines do exhibit a shorter baseline lifespan compared to the traditional UAS lines. This is likely due to the specific genetic background of the flySAM transgenic insertions, or a low level of "leaky" expression, as previously noted in the literature (Jia et al., 2018).

However, we believe that the lifespan extensions we observed for *DIP-β* flySAM is a robust biological effect, rather than an artifact of reduced viability for the following reasons. First, by utilizing the GeneSwitch (GS) system, we can compare the lifespan of flies with the exact same genetic background (+/- RU-486). This ensures that the extension we report is specifically due to the induction of the transgene, rather than a comparison between disparate lines with different basal fitness levels. Second, if the lifespan extensions merely represented a recovery from lower baseline viability, we would expect to see similar improvements across other flySAM lines in our screen. However, *DIP-β* was the only candidate across our screen that significantly increased lifespan in both sexes (Extended Data Figs. 7 & 8). Third, the lifespan-extending effect of *DIP-β* was independently confirmed using a traditional UAS-cDNA line, which importantly does not share the same baseline viability issues as the flySAM lines.

(c) Statistics: It is stated in the Methods that "statistical methods used are described in the figure legend of each relevant panel." However, there is no description of the statistics or sample sizes used in Figure 2.

We have updated the figure legends for Figure 2 to include the missing statistical details and sample sizes.

Specifically, for Fig. 2A: The reviewer is correct that with only two replicates of each time point (5d vs. 50d) in the initial proteomic screen, traditional p-value calculations lack the necessary power for meaningful interpretation. We have revised the legend to clarify that this panel represents a discovery-based screen. Candidates were selected based on biological relevance and specific enrichment thresholds to narrow the 872 proteins down to the 48 top candidates for screening (we were initially aiming to identify approximately 50 candidate genes for screening). For Fig. 2B: We have updated the legend to detail the parameters used for the Gene Ontology (GO) enrichment analysis.

(2) Figure 3: The authors use a glial GeneSwitch (GS) to knock down and overexpress candidate genes. In Figure 3A, they look at glial-GS>UAS-GFP with and without RU. Without RU, there is no GFP expression, as expected. With RU, there is GFP expression. It is expected that all cell body GFP signal should colocalize with a glial nuclear marker (Repo). However, there is some signal that does not appear to be glia. Also, many glia do not express GFP, suggesting the glial GS driver does not label all glia. This could impact which glia are being targeted in several experiments.

We thank the reviewer for this careful observation regarding the expression pattern of the *GSG3285-1* line and acknowledge that the overlap between this driver and the Repo-positive cells is not absolute.

Our selection of this specific GeneSwitch line was based on several critical experimental considerations: (1) To minimize background toxicity. We initially tested multiple *Repo-GeneSwitch* lines; however, we found they exhibited significant, genotype-dependent lifespan reductions upon RU486 administration, even in control crosses. This baseline toxicity confounded the interpretation of any potential lifespan effects. *GSG3285-1* was chosen for this study, as it provided a robust control baseline and didn’t show lifespan effects with RU486 treatment in multiple control lines. This is essential for lifespan studies. (2) The driver breadth and specificity. As noted in its original characterization (Nicholson et al., 2008) and a later study (Catterson et al. 2023), *GSG3285-1* is characterized as a pan-glial driver, though it may include a small population of sensory neurons. Furthermore, while *Repo* is a standard glial marker, its antibody does not label all glial subtypes with equal intensity. The "non-overlapping" signal observed in Figure 3A may reflect this staining bias. (3) The expression mosaicism. The fact that some glial cells do not show GFP expression suggests a degree of mosaicism, which is common to many GeneSwitch lines (Osterwalder et al., 2001). While we acknowledge this means our manipulations may target a broader subset — rather than every single glial cell — the fact that we still observed significant lifespan effects across two independent platforms (UAS and CRISPRa) suggests that the targeted population is sufficient to mediate these systemic effects.

We have added a clarifying statement to contextualize the choice of the *GSG3285-1* driver and its relationship to the Repo population.

(3) It is interesting that sex-specific lifespan effects were observed in the candidate screen.

(a) The authors should provide a discussion about these sex-specific differences and their thoughts about why these were observed.

We agree that the sex-specific effects observed in our lifespan screen are one interesting aspect of this study. We have added a dedicated section to the Discussion exploring these differences from both a technical and biological perspective.

On the technical side, the GeneSwitch inducer, RU486, can have sex-specific effects on metabolism and lifespan, depending on the nutritional environment (Dos Santos & Cocheme, 2024). Specifically, RU486 has been shown to counteract the lifespan-shortening effects of mating in females, an effect that is less pronounced in males (Landis et al., 2015; Tower et al., 2017). While we optimized our media and used the *GSG3285-1* line to minimize these baseline effects, it remains possible that certain genotypes exhibited a sex-specific sensitivity to the inducer itself. Beyond the technical considerations, sex differences in aging are well-documented in *Drosophila* and other organisms (Regan et al., 2016; Austad & Fischer, 2016). Male and female flies exhibit distinct transcriptional trajectories and metabolic shifts as they age. Furthermore, recent studies have highlighted that glial function and the neuroinflammatory landscape can differ significantly between sexes, which may dictate how a specific genetic manipulation impacts the aging process in a sex-dependent manner (PMID: 40951920). While our screen identifies *DIP-β* as a rare candidate that extends lifespan in both sexes, the prevalence of female-specific hits in our data suggests that the female "aging program" may be more plastic or responsive to the specific glial pathways we targeted. These observations provide a valuable foundation for future studies into the mechanisms of sex-specific neuroprotection.

(b) The authors should also provide information regarding the sex of the flies used in the glial cell surface proteome study.

It is a mixture of half male and half female flies. This information has been added to the main text, Fig. 1, and to the methods section.

(c) Also, beyond the scope of this study, examining sex-specific glial proteomes could reveal additional insights into age-related pathways affecting males and females differentially.

Agreed, this would be a great idea for future studies.

(4) The behavioral assay used in this study (climbing) tests locomotion driven by motor neurons. The proteomic analysis was performed with the adult brain, which does not include the nerve cord, where motor neurons reside. While likely beyond the scope of this study, it would be informative to test other behaviors, including learning, circadian rhythms, etc.

We thank the reviewer for this insightful point. While our initial proteomic screen focused on the adult central brain, our behavioral validation used a pan-glial driver, which targets glia throughout the entire nervous system, including the ventral nerve cord (VNC). We have addressed the reviewer's comment as below:

Additional behavioral data: As suggested, we performed Drosophila Activity Monitoring (DAM) assays to evaluate circadian locomotor rhythms in 50-day-old *DIP-β* overexpression flies compared to negative controls. Interestingly, we did not detect significant changes in circadian activity at this time point.

The difference between our climbing and circadian results highlights the complexity of age-related decline. In *Drosophila*, locomotor performance (i.e., climbing) and circadian coordination often decouple. For example, specific isoforms of human Tau (hTau) can induce severe cognitive and neurodegenerative deficits without affecting lifespan or motor coordination in the same manner (Sealey et al., 2017). Furthermore, motor-specific defects can emerge independently of systemic lifespan changes, as seen in certain SOD1 models of ALS (Hirth, 2010). It is possible that the 50-day timepoint represents a specific window where motor coordination is improved by *DIP-β*, while circadian circuits — governed by distinct glial-neuronal interactions — remain largely unaffected, or require a different temporal window for observation.

We agree that identifying the specific glial populations (central brain vs VNC) responsible for the improved climbing would be highly informative. While the current study establishes the pro-longevity effect of *DIP-β*, future work utilizing *in-situ* proteomics on the fully intact CNS (including the VNC) or specific VNC will be essential to map the stereotyped progression of these effects across the peripheral and central nervous systems.

(5) It is surprising that overexpressing a CAM in glia has such a broad impact on the transcriptomes of so many different cell types. Could this be due to *DIP-β* OE maintaining the brain in a "younger" state and indirectly influencing the transcriptomes? Instead of DIP-β OE in glia directly influencing cell-cell interactions? Can the authors comment on this?

We agree that the observed changes likely represent a combination of direct cell-cell interactions and a broader, more indirect maintenance of a "younger" physiological state.

Direct: Among the DIP family, *DIP-β* exhibits some of the strongest and most promiscuous binding affinities, interacting with a wide array of partners including Dpr6, 8, 9, 15, and 21 (Cosmanescu et al., 2018; Sergeeva et al., 2020). This biochemical flexibility allows *DIP-β* to potentially interface with a much broader range of neuronal subtypes than other DIP family members, such as DIP-δ, which exclusively binds Dpr12 and did not extend lifespan in our screen. It is possible that by overexpressing *DIP-β*, we may be partially compensating for the global downregulation of CAMs that typically occurs during aging, thereby preserving essential glial-neuronal communication integrity.

Indirect: By maintaining these primary glial functions and communication activities, *DIP-β* overexpression likely delays the overall "aging" of the brain. This preservation of neural health can have downstream effects on systemic physiology, such as the improved glia-fat body communication we observed in 50-day-old flies. In this model, the broad transcriptomic shifts are not necessarily all direct targets of *DIP-β*, but rather a signature of a brain that has successfully avoided the catastrophic breakdown of homeostasis typically seen in aged wild-type flies.

We have expanded the Discussion to clarify this distinction, adding that *DIP-β* likely acts as a "scaffold" or “bridge” for maintaining a younger brain state, which in turn preserves multi-organ communication.

**Reviewer #2 (Public review):**

This manuscript presents an ambitious and technically innovative study that combines in situ cell-surface proteomics, functional genetic screening, and single-nucleus RNA sequencing to uncover glial factors that influence aging in Drosophila. The authors identify DIP-β as a glial protein whose overexpression extends lifespan and report intriguing sex-specific differences in lifespan outcomes. Overall, the study is conceptually compelling and offers a valuable dataset that will be of considerable interest to researchers studying glia-neuron communication, aging biology, and proteomic profiling in vivo.

The in-situ proteomic labeling approach represents a notable methodological advance. If validated more extensively, it has the potential to become a widely used resource for probing glial aging mechanisms. The use of an inducible glial GeneSwitch driver is another strength, enabling the authors to carefully separate aging-relevant effects from developmental confounds. These technical choices meaningfully elevate the rigor of the study and support its central conclusions. The discovery of new candidate genes from the proteomics pipeline, including DIP-β, is intriguing and opens new avenues for understanding glial contributions to organismal lifespan. The observation of sex-specific lifespan effects is particularly interesting and warrants further exploration; the study sets the stage for future work in this direction.

At the same time, several areas would benefit from clarification or additional analysis to fully support the manuscript's claims:

(1) The manuscript frequently refers to "improved" or "increased" cell-cell communication following DIP-β overexpression, but the meaning of this term remains somewhat vague. Because the current analysis relies largely on transcriptomic predictions, it would be helpful to define precisely what metric is being used, e.g., increased numbers of predicted ligand-receptor interactions, enrichment of specific signaling pathways, or altered expression of communication-related components. Strengthening the mechanistic link between DIP-β, cell-cell communication, and lifespan extension, potentially through targeted validation of specific glial interactions, would substantially reinforce the interpretation.

We agree that a more precise description of “improved” or “increased” cell-cell communication is necessary.

Our conclusion that *DIP-β* overexpression is associated with “increased” cell-cell communication is based on the quantification of our CCC scores, which was performed using FlyPhoneDB2, a computational tool used to estimate cell-cell signaling from single-cell RNA-sequencing data (Liu et al., 2021; Qadiri et al., 2025). To infer cell-cell signaling, FlyPhoneDB2 and its predecessor, FlyPhoneDB, calculate “interaction scores,” comparing the expression levels of a curated list of ligand-receptor pairs between cell types (Liu et al., 2021; Qadiri et al., 2025). For example, if we detect a ligand in cell type A and its receptor in cell type B in *DIP-β* overexpression flies but didn’t detect both ligand and receptor in control flies, the CCC score is increased by 1. FlyPhoneDB2 additionally enables users to estimate signaling activity by also taking into consideration the expression of downstream reporter genes (Qadiri et al., 2025).

“Improved cell-cell communication” is our interpretation based on the CCC analysis. It is important to note that the metric being used here (increased CCCs) is the number of predicted ligand-receptor interactions, and that our CCC analysis was based entirely on inferences from snRNA-seq data. We have added further clarification to our manuscript, which now further expands on the results of our CCC analysis (i.e., the increased expression for 61% and decreased expression for 39% of ligand-receptor pairs we observed in our *DIP-β* overexpression group, compared to our negative control), which ultimately led us to conclude that *DIP-β* overexpression is associated with improved cell-cell communication.

(2) The lifespan screen is central to the paper, and clearer visualization and contextualization of these results would significantly improve the manuscript's impact. For example, Figure 3D is challenging to interpret in its current form. More explicit presentation of which manipulations extend lifespan in each sex, along with effect sizes and significance values, would provide clarity. Including positive controls for lifespan extension would also help contextualize the magnitude of the observed effects. The reported effects of DIP-β, while promising, are modest relative to baseline effects of RU feeding, and a discussion of this would help appropriately calibrate the conclusions.

We appreciate the reviewer’s suggestion to improve the clarity of the lifespan screen results. We have significantly revised Figures 3D, 3E, and 3F to provide a more intuitive summary of the candidate gene manipulations. Figures 3D and 3E now explicitly include the effect sizes and p-values for each candidate gene, broken down by sex. We also added a new Figure 3G with a visual layout that has been streamlined to allow for quick identification of manipulations that successfully extended lifespan.

The reviewer raises an important point regarding the use of positive controls to calibrate the magnitude of lifespan extension. We carefully considered adding a standard control (such as Rapamycin treatment); however, we opted against it for several methodological reasons:

As noted in the literature, the magnitude of lifespan extension from standard controls can vary drastically depending on genetic background and lab environment. For instance, Rapamycin-induced extension ranges from ~10% (Schinaman et al., 2019), to over 80% (Landis et al., 2024). We felt that adding a single positive control might provide a false sense of "calibration" rather than a true universal benchmark.

To ensure the robustness of our findings, we instead employed a dual-validation strategy. We confirmed the lifespan-extending effects of our candidates using both traditional UAS:cDNA and CRISPR-based overexpression. The fact that two independent genetic systems yielded consistent results provides strong internal evidence for the reported effects.

We acknowledge that the effects of *DIP-β* are modest when compared to the baseline impact of RU486 feeding. We have added a section to the Discussion addressing this. While the effects are subtle, their reproducibility across different overexpression platforms suggests they are biologically relevant, even if they do not reach the dramatic shifts seen in some caloric restriction or drug-based models.

We have further addressed this in the results section.

(3) Several figures would benefit from improved labeling or more detailed legends. For instance, the meaning of "N" and "C" in Figure 1D is unclear; Figure 3A should clarify that Repo is a glial marker; and Figure 5C appears to have truncated labels. Reordering certain panels (e.g., moving control data in Figure 4A-B) may also improve narrative flow. These refinements would greatly aid reader comprehension.

We have modified and improved the labeling of these figures to increase the clarity. For Fig. 1D, we added the explanation to the Figure legends. In brief, in the Tandem Mass Tag (TMT) isobaric labeling system, 128N is one of many channels (126, 127N, 127C, 128N, 128C, etc.) used to index and compare up to 18 samples simultaneously, improving throughput and reducing missing values.

Fig. 3A has been updated to clarify that Repo is the glial marker. Fig. 4A-D have been reordered so that the DIP- β lifespan results are presented before the control lifespan, which hopefully improves the narrative flow of this figure. The Fig. 4 references in the manuscript have also been updated to match these changes. Additionally, Fig. 5C has been updated to include the truncated x-axis and y-axis labels.

(4) A few claims would be strengthened by more specific references or acknowledgment of alternative interpretations. Examples include the phenoxy-radical labeling radius, the impact of H₂O₂ exposure, and the specificity of neutravidin. Additionally, downregulation of synapse-related GO terms may reflect age-related transcriptional changes rather than impaired glia-neuron communication per se, and this possibility should be recognized. The term "unbiased" to describe the screen may also be reconsidered, given the preselection of candidate genes.

These are good suggestions. We have added references for the phenoxy-radical labeling radius (Durojaye, 2021), the impact of H₂O₂ exposure (J. Li et al., 2021), and the binding specificity of neutravidin (J. Li et al., 2021). We have also removed the term “unbiased” from our manuscript.

Regarding the request to further address the downregulation of synapse-related GO terms, we believe this indicates a lack of clarity on our part. We did not intend to suggest that our GO analyses, which were based on our proteomics data, were necessarily indicative of impaired neuron-glia communication. Our conclusions regarding altered neuron-glia communication have come from our later snRNA-seq data and analyses. Inspired by this comment, we agree that our differential gene analysis may reflect transcriptional changes rather than impaired glia-neuron communication. We have added such alternative interpretation.

(5) Clarifying the rationale for focusing on central brain glia over optic-lobe glia would be useful.

Agreed! As the intended focus of this study was the more general changes occurring during normal brain aging, we chose to focus on the central brain for our glial cell-surface proteomics, which is responsible for most of the brain’s higher order functions, including learning and memory, signal integration, behavior, etc. As the optic lobes account for approximately half of all neurons in the adult *Drosophila* brain and are specialized to process visual stimuli (Robinson et al., 2025), we were concerned that including the optic lobes in our glial cell-surface proteomics could strongly bias our findings towards age-related changes in visual function, rather than the more general changes we intended to focus on. Such clarification has been added to the results section (Quantitative comparison of young and old proteomes).

**Recommendations for the authors:**

**Reviewer #1 (Recommendations for the authors):**

(1) Line 62: Can the authors expand on "several changes"?

We have added a sentence expanding upon this in the manuscript draft.

(2) Line 137: Can the authors provide a reference for the phenoxyl radical half-life?

Thanks for catching this. We’ve added our reference for the phenoxyl radical half-life.

(3) Figure 1B: The authors state that neutravidin stained glia; however, there is no glial marker (e.g., anti-Repo) in this panel.

We acknowledge the reviewer’s point. The lack of anti-Repo staining in Figure 1B is due to the requirements of the Neutravidin-Alexa 647 detection method. Because this procedure bypasses traditional primary and secondary antibody incubation to preserve the biotin signal, co-staining with Repo was not technically feasible. Nevertheless, we utilized the Repo-GAL4 driver to express UAS-CD2-HRP; since this driver is well-documented and specific to glial cells, the Neutravidin signal serves as a functional readout of the targeted glial population.

(4) Line 254: There is no Figure 2D.

We’ve corrected this to Fig. 2C.

(5) Lines 390-396: No reference to the respective figures.

We’ve made a couple corrections to reference all the respective figures.

(6) Figure 5C: The X-axis is cut off.

This has been corrected.

**Reviewer #2 (Recommendations for the authors):**

Minor inconsistencies (e.g., figure references-line 254 references "Figure 2D" where none exists) should be corrected.

We’ve corrected this to Fig. 2C.
